# Effect of *Piper sarmentosum* Extract on the Cardiovascular System of Diabetic Sprague-Dawley Rats: Electron Microscopic Study

**DOI:** 10.1155/2012/628750

**Published:** 2012-12-05

**Authors:** Zar Chi Thent, Teoh Seong Lin, Srijit Das, Zaiton Zakaria

**Affiliations:** ^1^Department of Anatomy, Faculty of Medicine, Universiti Kebangsaan Malaysia, Jalan Raja Muda Abd Aziz, 50300 Kuala Lumpur, Malaysia; ^2^Department of Physiology, Faculty of Medicine, Universiti Kebangsaan Malaysia, Jalan Raja Muda Abd Aziz, 50300 Kuala Lumpur, Malaysia

## Abstract

Although *Piper sarmentosum* (PS) is known to possess the antidiabetic properties, its efficacy towards diabetic cardiovascular tissues is still obscured. The present study aimed to observe the electron microscopic changes on the cardiac tissue and proximal aorta of experimental rats treated with PS extract. Thirty-two male Sprague-Dawley rats were divided into four groups: untreated control group (C), PS-treated control group (CTx), untreated diabetic group (D), and PS-treated diabetic group (DTx). Intramuscular injection of streptozotocin (STZ, 50 mg/kg body weight) was given to induce diabetes. Following 28 days of diabetes induction, PS extract (0.125 g/kg body weight) was administered orally for 28 days. Body weight, fasting blood glucose, and urine glucose levels were measured at 4-week interval. At the end of the study, cardiac tissues and the aorta were viewed under transmission electron microscope (TEM). DTx group showed increase in body weight and decrease in fasting blood glucose and urine glucose level compared to the D group. Under TEM study, DTx group showed lesser ultrastructural degenerative changes in the cardiac tissues and the proximal aorta compared to the D group. The results indicate that PS restores ultrastructural integrity in the diabetic cardiovascular tissues.

## 1. Introduction

Epidemiological studies show that the high prevalence mortality among diabetic patients is due to the cardiovascular complications that are involved in diabetes mellitus (DM). Data depict that more than 75% of all hospitalizations in diabetic subjects are attributed to the cardiovascular complications [1]. According to the Framingham study, it has been demonstrated that myocardial dysfunction in DM has no known etiological factors such as obesity, hypertension, coronary artery disease, hyperlipidemia, and alcoholism [2]. Although diabetic cardiac dysfunction is independent of any vascular compliance, it can be associated with degenerative vascular changes such as thickening of endothelium basal lamina and ultrastructural damages in the diabetic aorta [3, 4].

Chronic hyperglycemia is a complex, progressive disease which leads to increase in oxidative stress. The increase in oxidative stress results from increase in formation of free radicals and decrease in the antioxidant defense activity [5]. Experimental and clinical studies have suggested that diabetic state itself may be responsible for developing cardiac dysfunction and atherosclerosis [6]. 

The pathological features of diabetic cardiac dysfunction include reduction in transverse diameter of cardiomyocytes, thinning of myocardial fibers, and alteration in ultrastructural features of cardiomyocytes [3, 6]. All these features can be observed in type 1 DM, unlike type 2 DM [7]. Moreover, the degenerative changes can also be observed in great vessels like the aorta in DM [4]. The mechanisms' underlying cardiovascular complications are multifactorial. Endothelial dysfunction, endomyocardial fibrosis, arteriosclerosis, and direct toxic effects of hyperglycemia on heart and great arteries are present.

Depending on the incidences of DM and its complications, it is important to explore the new source of antidiabetic agents which are enriched with antioxidant properties. It is believed that the positive role of antioxidant activity will achieve good outcomes in DM and its long-term complications. The benefits of various herbs on the oxidative stress related disorders like DM and atherosclerosis have been well documented.

*Piper sarmentosum* (PS) is a traditional herb which is well-known for possessing antidiabeticproperties. PS is a creeping terrestrial herb which is cultivated in many countries like China, Borneo, Java, Thailand, and throughout the tropical lowlands of Malaysia. PS belongs to the family of Piperaceae [8]. It has been used to treat minor ailments such as headache, toothache, backache, waist pain, chest pain, muscle pain, menstrual pain, joint pain, cough, expectorants, and improves in urination, traditionally [9]. Phytochemically, the plant is rich in flavonoid compounds, phenylpropanoid (ascaricin, *α*-ascarone), phenolic compounds (xanthophylls, tannins) and calcium, iron, vitamin B_1,2_, C, E, *β*-sitosterol [10, 11]. Laboratory studies have been carried out to determine the antidiabeticrole of PS in streptozotocin-induced diabetic rats [12]. However, the cellular basis of improving heart tissues and aorta in DM following administration of PS extract has not been identified, to date.

The present study was aimed to determine the ultrastructural characteristics of experimentally induced abnormalities in the diabetic heart tissues and aorta. The study also aimed to explore the effect of PS supplementation on the cardiovascular damage.

## 2. Methods

### 2.1. Plant Material and Extract Preparation

The leaves of PS were purchased from Mentah Resources, Negeri Sembilan, Malaysia. The specimen was identified by Botanist from the Department of Botany, Universiti Kebangsaan Malaysia (Voucher Specimen-UKMB (No. 29851). The PS leaves were oven dried and grinded. Then Grounded PS powder was then mixed with 1L of water and boiled for 1 hr. It was then filtered, and the filtrate was sent for freeze drying to Forest Research Institute Malaysia (FRIM). PS extract was stored at 4°C for further use. During the treatment period, freeze-dried extract was dissolved in normal saline and administered orally at a dose of 0.125 g/kg body weight [12, 13].

### 2.2. Experimental Protocol

Prior ethical approval was obtained from the Institutional Animal Ethics Committees (UKMAEC). A total of thirty-two (*n* = 32) male Sprague-Dawley rats weighing between 200 ± 50 g were selected randomly for this study. The animals were kept in separate cages with a 12-hour light/dark cycle, and they were fed with standard rat chow and water *ad libitum*. Following 1 week of acclimatization period, the animals were equally divided into four groups (*n* = 8): untreated control group (C), PS-treated control group (CTx), untreated diabetic group (D), and PS-treated diabetic group (DTx). DM was induced by single injection of STZ (Sigma, USA) 50 mg/kg body weight, intramuscularly. Three days following DM induction, urine and the blood glucose levels were measured by Combur Test 10 M urine glucose strips (Roche Diagnostic, Australia) and ACCU-CHEK Advantage II glucometer with glucose strips (Roche Diagnostic, Australia), respectively. Rats with glucose present in urine were denoted as (+). Fasting blood glucose with 8 mmol/L or above were labelled as diabetic and taken for the study [14, 15]. Body weight, fasting blood glucose, and urine glucose levels were measured at 4-week interval throughout the study. PS extract (dose of 0.125 g/kg body weight) was administered for 28 days, following 4 weeks of DM induction [12]. At the end of the study, the rats were sacrificed and cardiac tissues and proximal aorta were collected for TEM study. 

### 2.3. Electron Microscopic Study

The left ventricular cardiac tissues and proximal aorta from two out of eight tissues per each group were collected according to a previous protocol [16]. Tissue was sectioned into 1 mm^3^ size with cross section and fixed with 2.5% glutaraldehyde for primary fixation. Approximately, 5–10 tissue slides per animal were used to investigate the ultrastructural findings. The specimen was examined using transmission electron microscope Tecnai G^2^ model at the voltage of 80 kV. (Philips, The Netherlands). All the electron microscopic findings were analysed by one Pathologist and one Anatomist in double-blinded fashion.

### 2.4. Statistical Analysis

Data was expressed as mean ± SD. Results were evaluated using three-way mixed ANOVA followed by Tukey's post hoc test. *P  *values  <0.05 (*P* < 0.05) were considered as significant. All statistical analysis was performed using SPSS statistical package version 19 (SPSS Inc., USA).

## 3. Results

### 3.1. Changes in Body Weight, Fasting Blood Glucose, and Urine Glucose

There were no significant differences in body weight, fasting blood glucose, and urine glucose, levels in all the groups prior to the STZ injection. However, a significant decrease (*P* < 0.05) in body weight was observed in the diabetic groups following 4 weeks of DM induction. However, body weight was significantly higher (*P* < 0.05) in the DTx group following 28 days of PS*  *administration compared to the D group (Table 1).

Fasting blood glucose and urine glucose levels showed significant increase in diabetic groups following four weeks of DM induction. Twenty-eight days following PS administration, all these parameters were found to be significantly decreased in DTx group compared to D group (Table 2). The changes in urine glucose level were also determined in DTx group after treating with PS extract (Table 3). 

### 3.2. Transmission Electron Microscopic Findings

#### 3.2.1. Cardiac Tissues

Under TEM examination, the cardiac tissues of the group D exhibited differences in subcellular morphology compared to C group. The characteristic differences easily observed were the condition of myocardial fibrils, gross deformation of mitochondria, and the gross invaginations in nuclei of the cardiomyocytes of D group. The protective role of PS extract against the ultrastructural features of heart tissues in DTx group was also observed. 

#### 3.2.2. Myofibrils

**Figure 1:**
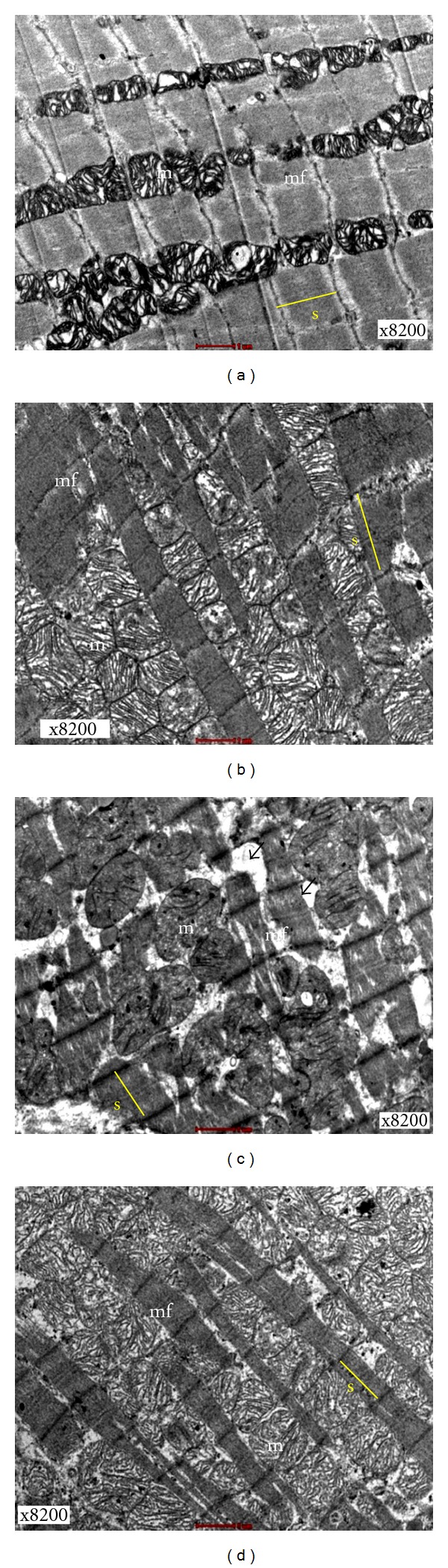
Transmission electron micrograph showing mitochondria (m) and myofibrils (mf) of the cardiac muscle tissues (x8200). (a) C group showing normal architecture of cardiac tissue, (b) CTx group showing normal architecture of cardiac tissue, (c) D group showing increase of mitochondria size, disarrangement of myofibrils, and patchy areas of cytoplasmic spaces (d) DTx group showing a normal architecture of cardiac tissue with less cytoplasmic spaces. Single headed arrows = cytoplasmic spaces, s = sarcomere.

Cardiac muscle fibres from C group and CTx group were abundant with regular arrays of myofibrils closely arranged within sarcomere (Figures 1(a) and 1(b)). This finding indicated that C group had a normal cellular morphology. However, sparse myofibrils within sarcomere (from Z line to Z line) were observed in D group (Figure 1(c)). Interestingly, these changes appeared to be less in DTx group. The cardiac muscle fibres in DTx group were less disturbed and less irregular arrays of myofibrils within sarcomere were examined (Figure 1(d)).

#### 3.2.3. Mitochondria

Overall, the mitochondria of cardiac tissues obtained from the left ventricle of C and CTx groups were typically intact and neatly arranged. There were a few cytoplasmic spaces, which were observed in the C and CTx groups (Figures 1(a) and 1(b)). The mitochondria in the D group showed an increase in size, compared to those in the C group. In addition, they were also disrupted. Furthermore, the increase in the patchy areas of cytoplasmic spaces was also observed (Figure 1(c)). Sections obtained from the DTx group showed a lesser extent of mitochondrial disruption and patchy areas of cytoplasmic spaces than the D group. Interestingly, the DM animals which received PS extract treatment for 28 days (DTx group) had intact mitochondria, reduction in mitochondria sizes and cytoplasmic spaces (Figure 1(d)). The ability of aqueous extract of PS to alter the subcellular morphological damages was clearly observed, in the present study. 

#### 3.2.4. Nuclei

**Figure 2:**
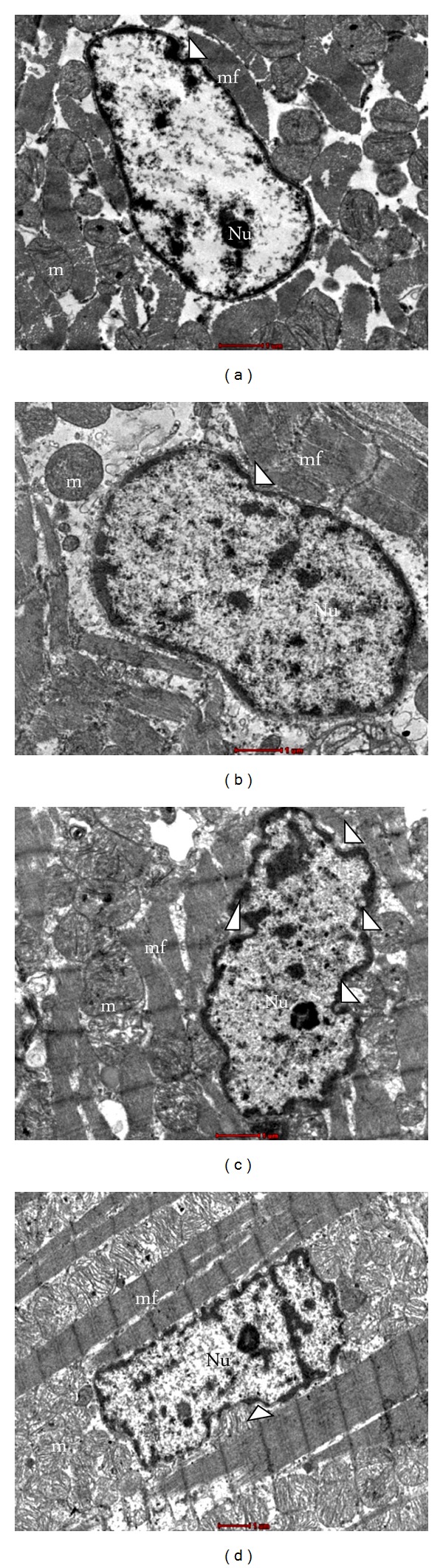
Transmission electron micrograph showing nucleus (N) of the cardiomyocytes in the cardiac muscle tissues (x8200). (a) C group showing normal nucleus of cardiomyocyte, (b) CTx group showing normal nucleus of cardiomyocyte, (c) D group showing presence of invagination in nucleus of cardiomyocyte, (d) DTx group showing less invagination in nucleus of cardiomyocyte. m = mitochondria, mf = myofibrils, Nu = nucleolus, white arrow heads = invaginations of cardiomyocyte nucleus.

Normally, nuclei from the cardiomyocytes were found to have few invaginations. Invaginations were defined as being at least 0.3 *μ*m in depth and less than 1 *μ*m in width according to the previous study [3]. However, in our recent work, only the visual appearances of invaginations were being focused. C and CTx groups showed only a few invaginations as normal morphological features (Figures 2(a), 2(b)). The specimens from untreated D group showed more invaginations with deformation of the nucleus (Figure 2(c)). DTx group was also found to possess few invaginations in their nuclei. There were lesser invaginations in the DTx group compared to D group. The nucleus reverted to its normal shape (Figure 2(d)). These results showed out that the morphological features of the cardiac tissues in STZ-induced DM rats were damaged. Perhaps, the administration with PS extract could improve these degenerative changes that occurred in cardiac tissues of DM rats. No differences in the extent and visual sizes of nucleoli were observed in all the groups.

#### 3.2.5. Proximal Aorta

**Figure 3:**
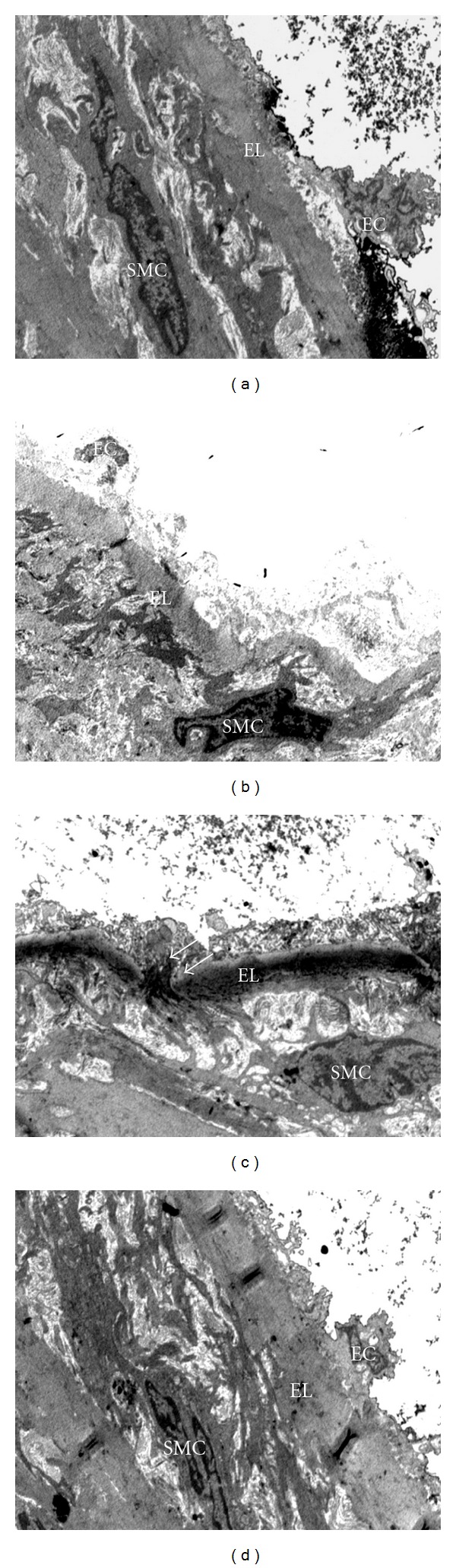
Transmission electron micrograph showing the ultrastructural organization of the proximal aorta (x4200). (a) C group showing normal ultrastructural organization of aorta with the presence of endothelial cells (EC), (b) CTx showing normal ultrastructural organization of aorta with the presence of endothelial cells (EC), (c) D group showing disruption of elastic lamina (EL), proliferation of smooth muscle cells (SMC), and migration of smooth muscle cells (single headed white arrows), (d) DTx group showing less degenerative changes of proximal aorta with the presence of endothelial cells (EC).

In group C, the proximal aorta showed normal appearance of endothelial cells in elastic lamina, the elastic lamina was also intact and presence of smooth muscle cells in TM layer was observed (Figure 3(a)). Similar findings were also observed in CTx group of rats (Figure 3(b)).

**Figure 4:**
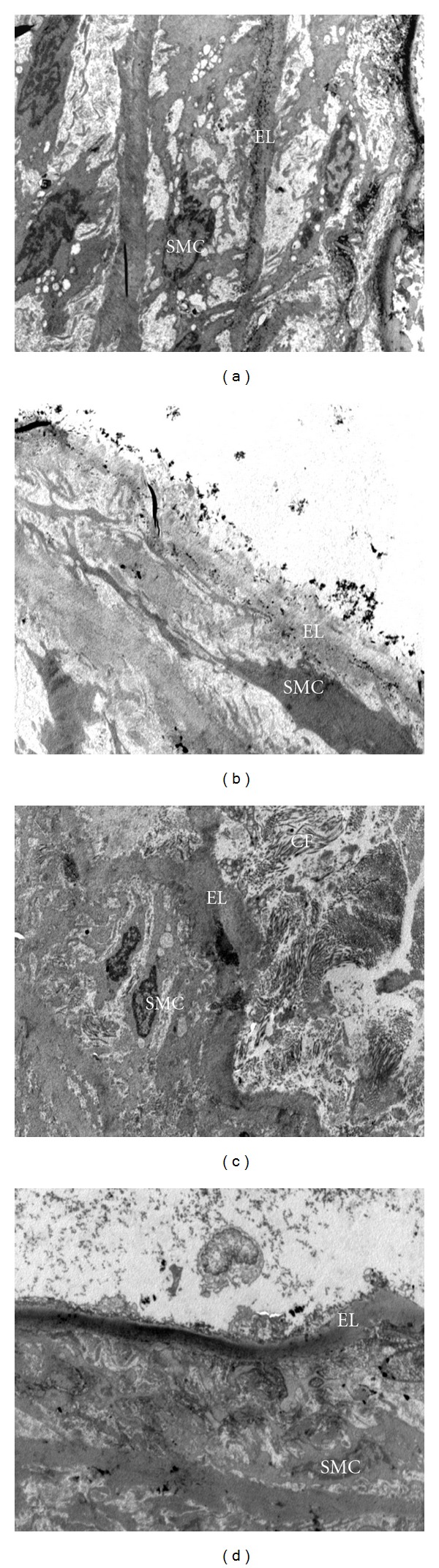
Transmission electron micrograph showing the ultrastructural organization of the proximal aorta (x2550). (a)**  **Control (C) group showing normal ultrastructural organization aorta, (b) Control group supplemented with PS extract (CTx) showing normal architecture of proximal aorta, (c) Nontreated diabetic (D) group showing disruption of elastic lamina (EL), presence of collagen fibres (CF), (d) Diabetic group supplemented with PS extract (DTx) showing restoration of ultrastructural organization of proximal aorta.

However, D group showed disruption in the elastic lamina whereby the endothelial cells were difficult to observe under TEM. In many parts of the elastic lamina, there was loss of endothelial cells. That may be due to the disruption of elastic lamina in aortic wall of D group rats. It was found that the smooth muscle cells in TM layer were migrating into elastic lamina breaking through the elastic lamina. Furthermore, the proliferation of smooth muscle cells was also observed in the D group. The morphological changes observed in endothelial cells changes indicated endothelial injury (Figure 3(c)). There were deposits of collagen fibres in the DM groups (Figure 4(c)). On the other hand, DTx group showed lesser degenerative changes compared to the D group. Decrease in the disruption of the elastic lamina, decrease in the smooth muscle cells proliferation, and the presence of the endothelial cells were some of the notable findings which were observed in DTx group (Figure 3(d)). These results showed that the ultrastructural organization of aorta was disturbed in DM rats. It was considered to be early development signs of atherosclerosis in experimental-induced DM rats. Administration with PS extract in the DM rats showed reduction in damages and restored the ultra- structural organization of the proximal aorta. The detailed ultrastructural changes of proximal aorta in both control and DM rats were observed. The D group showed presence of collagen fibres compared to the C group (Figures 4(a), 4(b), 4(c), and 4(d)). 

## 4. Discussion

In the present study, body weight in DM rats was significantly decreased compared to C group. Such a significant weight loss has been observed in previous work using experimentally induced DM animals [17]. The reason behind this is attributed to the metabolic disturbances that occurred in DM, in which calorie deprivation plays a crucial role. Calorie deprivation leads to decrease in protein synthesis and promotes protein degradation in longer-lasting DM state [2]. However, treatment with 0.125 g/kg of PS extract showed improvement in the body weight in DM. 

The increase in body weight following administration of PS extract in DM group was not clearly explained in earlier studies. Perhaps, we may hypothesise that the plant extract supplied the body with the essential antioxidant nutrients and vitamins. These were needed to enhance the immune system, eliminating the free radicals and to keep the oxidative stress in balance. The leaves of PS were reported to maintain energy, general ability, and fitness [8]. In fact, the quercetin (flavonoid compounds) present in PS has been marketed in the United States as a dietary supplement with the recommended daily doses of 200–1200 mg and it was published as an effective food of DM patients [18, 19]. These reasons could explain why PS leaves improved the body weight in the experimental DM rats. 

The fasting blood glucose and the urine glucose level in the present study were significantly high in DM rats compared to the control group (*P* < 0.05). These findings are similar to the previous reports, which showed that following administration of STZ, the symptoms of hyperglycaemia and glycosuria appeared in the animals [15]. It is well known that induction of STZ can cause irreversible damage of *β* cells of the pancreas, reduction of insulin secretion, and thereby leading to increase in blood glucose level. STZ impairs glucose oxidation and decreases insulin biosynthesis and secretion [20]. In order to confirm, additional urine glucose level was checked in all groups of rats during the experiment. DM rats showed increased in urine glucose level of (++) to (+++). Similar findings were also published in a previous study [15]. However, the administration with PS extract showed hypoglycaemic effect on both control and DM rats. This is in accordance with the previous findings which showed that repeated oral administration of 0.125 g/kg and 0.25 g/kg of aqueous extract of PS for 7 days could reduce the blood glucose level both in control and DM rats. In fact, the dose 0.125 g/kg of PS employed in the present work was adopted from a previous study [12]. Moreover, the crude extract of PS was also shown to decrease blood glucose level in alloxan-diabetic rabbits [21].

The mechanism modifying the state of chronic hyperglycaemia depends on the phytochemical compounds that present in the leaves of PS. It has been postulated that PS is enriched with tannins, hydrocinnamic acid, *β*-sitosterol, pellitorine, pyrrole amine, sarmentine, and sarmentosine [22]. Tannins are the active potent antioxidant compounds that have beneficial effects on DM. The compounds contain an enzyme: a benzoic acid related molecule inhibited insulinase which could enhance the insulin secretion thereby inhibiting insulin degradation [23]. Importantly, PS has shown the presence of antioxidant compounds like quercetin. Quercetin has a positive role in decreasing blood glucose levels, promoting the regeneration of the pancreatic islets, and increasing insulin release with high superoxide scavenging activity in DM rats [24]. 

Electron microscope is a precious tool for examining the ultrastructural pathology in the progression of DM. It can be used to tie the subcellular changes to some of the biochemical, functional, and histological changes determined in the DM affected tissues. Earlier studies have mentioned that DM induced several visible and measurable changes in the ultrastructure of the cardiomyocytes [3]. Qualitatively, the myofibrils were disordered, the mitochondria were disrupted, and interstitial collagen deposits were found in the left ventricle of D group in the present study. There were also alterations in the diabetic cardiomyocytes nuclear morphology including the increase in presence of invaginations compared to the C group and CTx groups. In the present study, the changes on the left ventricle were evaluated after 8 weeks of STZ induction, which is more susceptible to DM induced pathology, according to the past reports [3].

The disordered myofibrils observed in untreated D group were well observed. These findings are consistent with previous reports that also showed loss of myofibrils in the diabetic cardiomyocytes. In addition, the researchers pointed out that there were no changes in sarcomere length related to DM [25]. However, the sarcomere length was not measured in the present study. DM induced changes in the mitochondria, cytoplasmic area were consistent with the previous findings. The observation of patchy mitochondrial disruption is a key characteristic feature that has been reported in various studies. The present findings are similar to past researches which described the presence of invaginations in nuclei of the cardiomyocytes of DM rats [26]. Moreover, in one of our recent studies, it was shown that PS is competent to maintain the histological integrity of both cardiac tissues and the proximal aorta [27].

The mechanism of subcellular changes shown in diabetic hearts is still not well understood. It has been considered that DM is associated with an abnormality in myocardial energy metabolism which leads to reduce glucose transport, increase in FFA metabolism, and reduced ATP production. Metabolism shifts from using plasma glucose to the excess use of FFA, thereby producing glycogen, and this causes myocardial injury. The mitochondria deformation may be probably due to increase fragility of the mitochondrial membrane as a result of DM [28]. It has been mentioned earlier that the primary site of damage in DM is the heart which contributes a large number of mitochondria [28].

Regarding the cardiac dysfunction in experimentally induced DM rats, several authors reported that metabolic deteriorations in DM induce cardiac structural alterations as well as biochemical changes. Eventually, it was shown that hyperglycaemia causes hyperosmolarity, shrinking of ventricular cardiomyocytes, which deform the nuclear morphology and mitochondrial disruption which in turns leads to disorganization in myofibrils as seen under TEM. 

There is paucity of studies on the protective role of herbs or traditional medicines with regard to diabetic cardiac dysfunction. To the best of our knowledge, the present study was the first of its kind to observe the electron microscopic findings on the protective role of PS extract in DM. However, the action of PS extract in preventing ultrastructural damages of diabetic cardiac tissues was not fully understood. This study demonstrated that 28 days of PS extract are sufficient to restore some of the ultrastructural damages revealed in cardiac tissues of DM rats. Interestingly, the descriptive appearance of mitochondrial, the cytoplasmic area, the collagen fibres, and the presences of invaginations in nuclei of cardiomyocytes were reverted. The antioxidant activity of quercetin would be helpful to manage glucose uptake and increased levels of mitochondrial reactive oxygen species (ROS) linked to hyperglycaemia [9, 24]. This would prevent the cardiac tissues damages by inhibiting the metabolic disturbances of DM. Narigenin is known to improve endothelial function that reduces the risk of developing coronary heart disease [8]. Eventually, from the present studies, it was proved that repeated oral administration with PS extract can restore the ultrastructural integrity of diabetic cardiac tissues.

Regarding the TEM findings of aorta in the present study, the ultrastructural organization of proximal aorta was disturbed in DM rats. In the present study, the qualitative disruption of elastic lamina indicated the endothelial injury. In addition, the migration of smooth muscle cells from medial layer to intima layer was also observed. These findings were considered to be early events in the development of the atherosclerotic lesion in DM. In the present study, the proximal aorta (approximately 2 cm) was used as most of the hemodynamic changes could be occurred in this area [29]. The present results are similar to previous findings which reported that induction of DM with STZ can cause vascular wall damage [4].

Unfortunately, to date, the underlying mechanisms of the ultrastructural disorganization of the aorta in DM have not been explored yet. The increase in oxidative stress and the accumulation of advanced glycation end products (AGEs) are considered as the predisposing factors of the atherosclerosis [29]. The interaction of AGEs with their specific receptors (RAGE) on endothelial cells results in reduction of endothelial barrier function. This leads to increase endothelial permeability and subsequently results in transendothelial migration. Even in smooth muscle cells, binding of AGEs modified protein to RAGE is associated with increased cellular proliferation. The increasing AGEs accumulation in vascular walls facilitates the migration of inflammatory cells. Moreover, in hyperglycaemia the increase in free radical damage like ROS may accelerate the atherosclerotic reaction by producing superoxide. It was also proved that there was a strong link between glycoxidation product and the severity of diabetic retinal, renal, and vascular diseases [30]. Therefore, the disturbances of endothelial cells and the smooth muscle cells proliferation were observed as endothelial dysfunction in DM rats. Previous studies proved that hyperglycaemia, hyperlipidemia, and high oxidative stress might cause morphological alterations in the aorta and it was considered to be the important factor in initiating the atherosclerotic lesions in type 1 DM [4].

PS, which has been used as a nutritional supplementation, has several potential benefits including therapeutic potential and capable of scavenging free radicals. Laboratory studies have shown that PS*  *possesses high antioxidant efficacy as it shows 87.6% of superoxide free radical scavenging activity, 98% of lipid peroxidation inhibitory activity, and 96% of radical scavenging activity [8]. All of these activities have had a positive impact on the DM and its complications. Antiangiogenic activity on experimental rabbits fed with cholesterol diet of aqueous extract of PS has been reported earlier [13]. It was believed that PS could modify the cell structure against damages as it can improve the hyperglycaemic condition. Therefore, the mechanism by which PS supplementation exerts vascular morphology is probably due to its antioxidant properties.

## 5. Conclusion

Various studies have highlighted the role of herbs in preventing the oxidative stress disorders like DM. According to the present study, PS extract proved to have positive effect on body weight, fasting blood glucose level, and urine glucose level in experimentally induced DM rats. Moreover, under electron microscope, it was found out that the supplementation with PS extract produced less subcellular changes in diabetic cardiovascular tissues by reducing the cardiac dysfunction and early signs of atherosclerosis. Large-scale studies are needed to further define the potential effects of PS extract.

## Figures and Tables

**Table 1:** Table showing changes in the body weight (gm) of rats in the various groups throughout the study period.

Groups	Week 0	Week 4	Week 8
(1) C group	219 ± 8.91	278 ± 5.02	310 ± 5.34
(2) CTx group	224 ± 10.13	276 ± 5.22	345 ± 7.43
(3) D group	232 ± 8.24	193 ± 10.51	178 ± 10.91*
(4) DTx group	237 ± 9.41	194 ± 10.43	231 ± 13.52*

Data were expressed as mean ± SD. ^∗^ *P* < 0.05 significant decrease compared to control (C).

C represents control group supplemented with normal saline, CTx represents control group supplemented with PS extract; D represents diabetic group supplemented with normal saline; DTx represents diabetic group supplemented with PS extract.

**Table 2:** Table showing changes in the fasting blood glucose level (mmol/L) of rats in the various groups throughout the experimental period.

Groups	Week 0	Week 4	Week 8
(1) C group	5.1 ± 0.21	5.1 ± 0.33	5.0 ± 0.33
(2) CTx group	5.4 ± 0.53	5.2 ± 0.22	3.0 ± 1.21
(3) D group	4.9 ± 0.32	30.7 ± 1.94	31.9 ± 1.72*
(4) DTx group	5.3 ± 0.41	31.7 ± 1.64	23.2 ± 2.24*

Data were expressed as mean ± SD. ^∗^ *P* < 0.05 significant decrease compared to control (C).

C represents control group supplemented with normal saline, CTx represents control group supplemented with PS extract; D represents diabetic group supplemented with normal saline; DTx represents diabetic group supplemented with PS extract.

**Table 3:** Table showing changes in the urine glucose level of rats in the various groups throughout the experimental period.

Groups	Week 0	Week 4	Week 8
(1) C group	(−)	(−)	(−)
(2) CTx group	(−)	(−)	(−)
(3) D group	(−)	(++)	(+++)
(4) DTx group	(−)	(++)	(+)

In the table, (−) represents absence of glucose; (+) represents presence of glucose; (++) represents high level of glucose, and (+++) represents higher level of glucose in the urine.

In the table, C represents control group supplemented with normal saline; CTx represents control group supplemented with PS extract; D represents diabetic group supplemented with normal saline; DTx represents diabetic group supplemented with PS extract.
